# Temporal dynamics of antennal responses encode plant stress and ecological relevance in *Spodoptera littoralis* moths

**DOI:** 10.1016/j.isci.2026.117941

**Published:** 2026-10-22

**Authors:** Guy Zer Eshel, Neta Shvil, Michael Davidovitz, Dana Ment, Yossi Yovel

**Affiliations:** 1 School of Zoology, George S. Wise Faculty of Life Sciences, Tel Aviv University, Ramat Aviv, Tel Aviv-Yafo 6997801, Israel; 2 Sagol School of Neuroscience, Tel Aviv University, Ramat Aviv, Tel Aviv-Yafo 6997801, Israel; 3 School of Mechanical Engineering, Tel Aviv University, Ramat Aviv, Tel Aviv-Yafo 6997801, Israel; 4 Plant Protection Institute, Agricultural Research Organization - Volcani Institute, Derech Maccabim 68, Rishon LeZion, Israel; 5 The Steinhardt Museum of Natural History and Israel National Center for Biodiversity Studies, Tel Aviv University, Ramat Aviv, Tel Aviv-Yafo 6997801, Israel

**Keywords:** insect olfaction, *Spodoptera littoralis*, electroantennogram, EAG, machine learning, sexual dimorphism, chemical ecology, peripheral processing, plant volatile coding

## Abstract

Polyphagous insects draw fitness-relevant information from complex plant odor blends, yet electroantennography (EAG) studies often emphasize response amplitude rather than temporal structure. We combined natural plant volatile stimuli, temporal EAG analysis, and machine-learning classification to examine the responses of male and female *Spodoptera littoralis* antennae to host/edible plants, non-hosts, and drought-stressed plants. EAGs entailed much information about plant volatiles. Female responses showed stronger functional organization and higher classification performance for the above-mentioned categories, particularly for non-host plants, whereas males retained discrimination only under high-contrast stress conditions. These findings suggest that peripheral antennal dynamics preserve biologically relevant information about plant suitability and physiological state. More broadly, this framework offers a new approach for linking peripheral olfactory physiology with future electrophysiological and behavioral experiments.

## Highlights

• Temporal moth EAG dynamics encode information in natural plant odor blends
• Unsupervised analyses reveal ecological organization of antennal odor responses
• Supervised classifiers decode plant ecological category and stress state
• Females show stronger ecological and drought-stress decoding than males

## Graphical abstract

**Figure:**
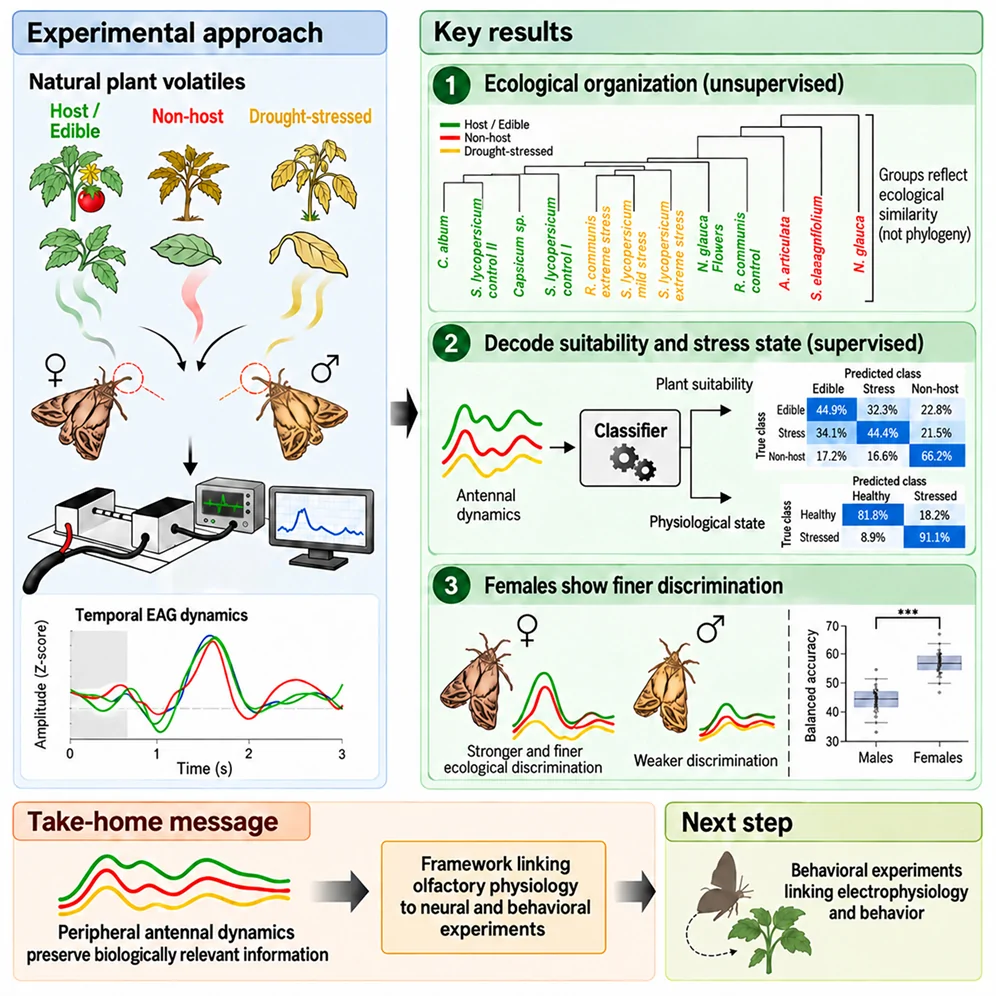

## Teaser

Entomology; Animal physiology; Sensory neuroscience; Machine learning

## Introduction

Sensory evolution is shaped by life-history trade-offs,^1^ necessitating sensory systems capable of extracting biologically relevant signals from complex environments.^2^^,^^3^^,^^4^ This task is further complicated by the stage-dependent nature of chemical information: a volatile compound that signals a profitable energy source for an adult may be irrelevant, or even harmful, when evaluating a site for offspring development. Thus, the value of a chemical cue is not universal, but contingent on the ecological demands of the organism’s current life stage.^5^^,^^6^^,^^7^^,^^8^ This tension between self-maintenance and reproductive investment is a central theme in behavioral ecology, yet the sensory mechanisms that enable such finely tuned, context-dependent decision-making remain a subject of study.^9^^,^^10^^,^^11^^,^^12^

In a wide range of taxa, this evolutionary tension manifests as sex-specific sensory specializations. While both sexes must navigate olfactory landscapes to satisfy metabolic requirements (foraging), females are subject to the additional selective pressure of identifying optimal oviposition sites, a process requiring a sensory architecture that reflects the larvae-specific ecological requirements.^13^^,^^14^^,^^15^ This task is complicated by the substantial overlap among volatile compounds emitted by different plants. Phytophagous insects possess antennae equipped with olfactory sensory neurons (OSNs) and receptors (ORs) specialized to detect Green Leaf Volatiles (GLVs); primarily alcohols, aldehydes, and esters that serve as near-universal constituents of angiosperm foliage.^16^^,^^17^^,^^18^^,^^19^^,^^20^ Given this significant compositional overlap between host and non-host plants, and the fact that many insect ORs are broadly tuned,^21^ the sensory system must perform a sophisticated classification to distinguish subtle ecological signals from the background of frequent volatiles.^4^

This need to extract behaviorally relevant information from chemically complex environments raises a methodological challenge: how can we measure not only whether an insect detects plant odors, but also how odor information is dynamically represented at the sensory periphery?

Historically, our understanding of these olfactory inputs has often relied on Electroantennography (EAG) to capture the summed electrical activity of olfactory sensory neurons (OSNs).^22^ While head-space EAG has advanced the field by using natural plant volatile blends, thereby preserving naturally co-occurring odorants and variation in their relative abundance, traditional analyses of these signals have remained limited.^23^^,^^24^ Most studies continue to prioritize a single parameter such as the peak amplitude, thereby overlooking the complex temporal dynamics and morphological information embedded within the signal, which reflects the variation in spatiotemporal responses of the antennal receptors.^25^ Moreover, the overlap in GLV composition among different plants makes it more likely that response intensity alone would produce similar or ambiguous signals across plant species. Contemporary analytical frameworks are therefore shifting toward integrative signal processing, utilizing robust computational architectures to facilitate a more comprehensive classification of these high-dimensional biological responses.^26^^,^^27^^,^^28^^,^^29^

In this study, we examined the odorant space of *Spodoptera littoralis*, a generalist pest posing a significant global threat to tomato production. Its pest status is closely linked to opportunistic feeding and oviposition habits, which allow it to locate, evaluate, and exploit diverse host plants across variable ecological conditions.^30^ The olfactory system of *S. littoralis* has been extensively characterized, serving as a model for studying higher-order sensory processing. Key features include specialized sensitivity to herbivore-induced plant volatiles and pheromones, sexually dimorphic neural architecture, and robust neuronal plasticity.^31^^,^^32^^,^^33^ Additionally, the antennae of both sexes were found to exhibit comparable physiological response magnitudes and sensitivities to a diverse range of plant volatiles.^32^

Here, we stimulated the *S. littoralis* antennae with natural volatiles from various healthy and stressed plant tissues to reconstruct its ecologically relevant chemical landscape. By using temporal alignment, clustering, and machine learning, we examined how EAG signal structure encodes ecological information and enables context-dependent classification at the antennal level.

## Results

We quantified the antennal responses of male and female *Spodoptera littoralis* (continuously reared on castor beans) to plant-derived volatile cues of varying ecological relevance using electroantennograms (EAG) (Figures 1A–1C). In all assays, volatiles from excised plant tissues were delivered simultaneously to both antennae. Because odor intensity could not be directly standardized across plant species and treatments, all EAG signals were *Z* score normalized. This normalization ensured that subsequent analyses focused on the temporal shape of the antennal response rather than absolute amplitude.

**Figure 1:**
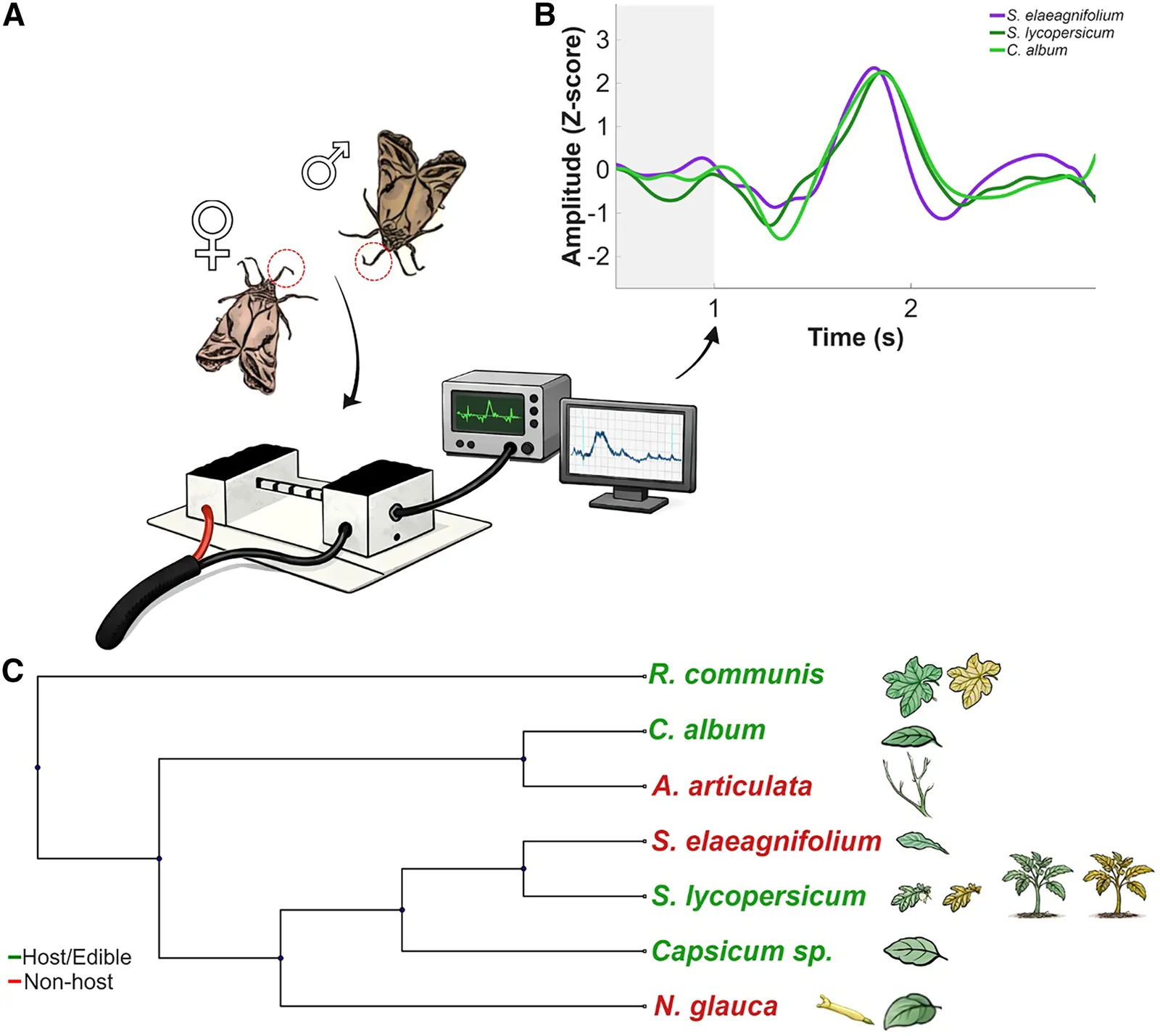
Experimental workflow for characterizing the olfactory landscape of *Spodoptera littoralis* (A) Schematic of the electroantennography (EAG) setup using adult male and female moths as biological sensors. Excised antennae were exposed to plant-derived volatile organic compounds (VOCs), and the resulting depolarization potentials from olfactory receptor neurons were recorded. (B) Representative post-processed EAG signals elicited by volatiles from three distinct plants. Gray-shaded area represents the 0.5 s odor-stimulation period. (C) Phylogenetic relationships among the seven plant species selected for volatile screening, representing the families Solanaceae, Chenopodiaceae, and Euphorbiaceae. Species names in green denote host/edible plants, while red indicates non-host species. Right: morphological depictions of the plants and specific treatments used for odor collection. Whole plants show mildly stressed (yellow) vs. control (green) tomato leaves. Other healthy plant volatiles are shown in green; drought-stressed plants are indicated by pale/yellow-green leaves. *Nicotiana glauca* flowers are marked by the yellow *N. glauca* flower icon. The plant species presented are: *Ricinus communis*, *Chenopodium album*, *Anabasis articulata*, *Solanum elaeagnifolium*, *Solanum lycopersicum*, *Capsicum* sp., and *Nicotiana glauca*.

We evaluated antennal sensitivity across seven plant species (Figure 1C). These were assigned *a priori* to biologically motivated groups: host/edible plants were classified based on documented larval performance and previous rearing observations of the focal species^30^ and were tested under both hydrated and drought-stressed conditions. *Nicotiana glauca*, *Solanum elaeagnifolium*, and *Anabasis articulata* were classified as putative non-host plants based on previous rearing experience and published evidence of insecticidal, antifeedant, or larvicidal activity.^34^^,^^35^^,^^36^ Because this categorization was based on behavioral studies involving various insect species and exposure methods, the grouping should be interpreted as biologically motivated rather than chemically homogeneous. Each stimulus was presented in triplicate to every antennal preparation to assess response consistency.

### Unsupervised hierarchical clustering

To identify the intrinsic organization of EAG dynamics, we applied unsupervised hierarchical clustering using derivative dynamic time warping (DDTW) on normalized traces. Consensus dissimilarity matrices were generated using median pairwise values to minimize outlier bias. The resulting dendrogram, validated via the cophenetic correlation coefficient, demonstrated high structural robustness across both sexes through bootstrap resampling (1,000 iterations; females: *n* = 54, *r* = 0.87; males: *n* = 36, *r* = 0.83) (Figures S1A and S1B). This stability was further supported by adjusted rand index (ARI) and k-nearest-neighbor metrics, which significantly exceeded permutation-based null expectations (*p* = 0.001, AUC) (Figures S1C and S1D). Importantly, this EAG-based organization did not simply recapitulate plant phylogenetic relatedness, as tested later in discussion.

Hierarchical clustering revealed a distinct, sex-specific organization of plant odor representations (Figures 2A and 2B). To determine if these physiological patterns reflect plant evolutionary history, we compared sex-specific EAG distance matrices to the phylogenetic distance matrix using the Mantel test. For both sexes, EAG-based organization was decoupled from phylogeny, with no significant correlations detected (Mantel test; females: *r* = −0.12, *p* = 0.78; males: *r* = 0.09, *p* = 0.79; *n* = 7 taxa; Figures 2A–2C). Given the divergence in dendrogram topology between sexes, we tested whether the functional organization of odor similarity was conserved according to ecological relevance. Odors were grouped into three categories: Non-host, Stressed, and Host/Edible.

**Figure 2:**
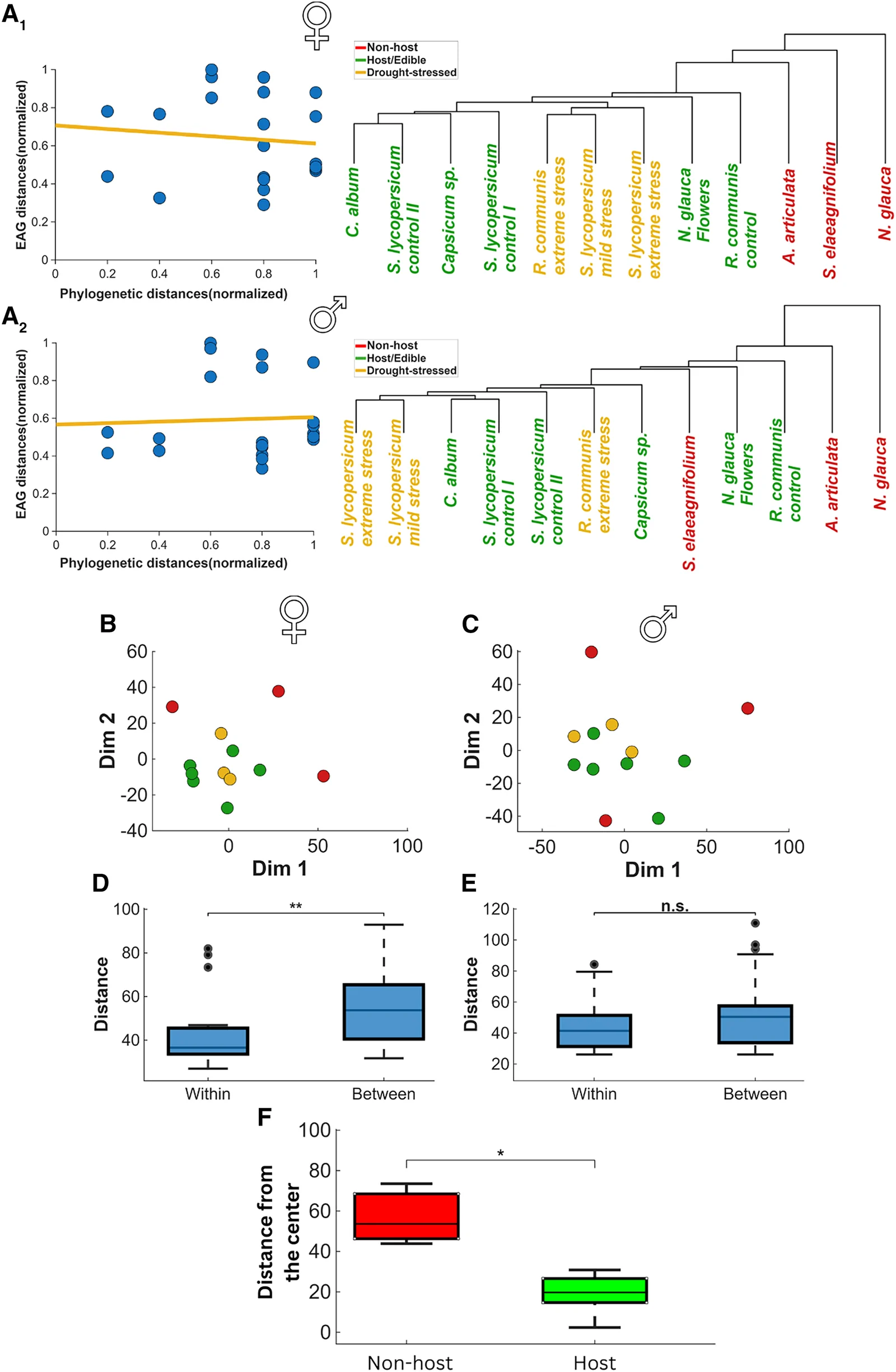
Sex-specific functional organization of antennal plant-odor representations in *S. littoralis* (A) Hierarchical clustering of electroantennogram (EAG) responses for females (A_1_) and males (A_2_). Colors denote ecological categories: non-host (red), host/edible (green), and drought-stressed (yellow). Left: Correlation between normalized EAG physiological distances and normalized phylogenetic distances. Right: Dendrograms representing odor-based similarity. (B and C) Non-metric multidimensional scaling (nMDS) ordinations of odor similarity for females (B) and males (C). Points are colored by ecological category. Both sexes show substantial overlap in plant taxa odor space, as reflected by mean silhouette values near zero (females: *S* = −0.01; males: *S* = −0.05). (D and E) Comparison of within-ecological-category versus between-ecological-category distances for (D) females and (E) males. Females exhibit a significant median compression (31.9%) of within-category nMDS distances relative to the global space (KS test, *n* = 54, *p* = 0.002). This functional clustering is significantly weaker and non-significant in males (17.8% compression; KS test, *n* = 36, *p* = 0.12). (f) Pooling males and females together and comparing the distance from the center of the odor space between host and non-host groups (Welch *t* test, *n* = 90, *p* = 0.04). Boxplots in (d–f) show the median, interquartile range (IQR), and 1.5 × IQR whiskers. Statistical significance is indicated as ∗*p* < 0.05, *p* < 0.01; n.s., not significant.

Odor distances were significantly smaller within categories than between categories, though the magnitude of this effect was sex-dependent. This distinction was significant in females but not in males (KS test, *p* = 0.002 and *p* = 0.12, respectively, Figures 2D and 2E). Nevertheless, non-host volatiles were clustered on the periphery of the odor space in both males and females (Welch *t* test, *n* = 90, *p* = 0.04, Figure 2F).

### Supervised classification

To evaluate sex-specific differences in volatile perception, we employed a Gradient-Boosting classifier within a “leave-one-individual-out” cross-validation framework. Initially, we grouped plant volatiles into the three functional categories defined previously (Non-host, Stressed, and Host/Edible) and filtered the dataset for signals with the highest signal-to-noise ratios while maintaining the same sample sizes (*n* = 54 females, *n* = 36 males). To account for group imbalances, we performed 100 iterations of Random Under-sampling to ensure equal group sizes (*n* = 36). We then tested the model’s ability to decode the ecological category of a stimulus based on the antennal signals of each sex. Under this procedure, performance diverged significantly between the sexes: while males’ accuracy did not differ significantly from the null distribution (males = 43.3 ± 3.1%, permutation test, *p* = 0.06, Figure 3A), female performance was significantly above chance (54.47 ± 2.12%, permutation test, *p* = 0.002, Figure 3B). Consequently, females had a significantly higher probability of correct classification than males (generalized linear mixed-effects model [GLMM], *p* = 0.002; Figure 3C). When comparing classifier performance with a simple one-parameter area-under-the-curve (AUC)-based classifier, we found that the balanced accuracies of the AUC-based classifier did not differ significantly from chance for either sex (females: 34.1%; males: 33.8%; Figure S2).

**Figure 3:**
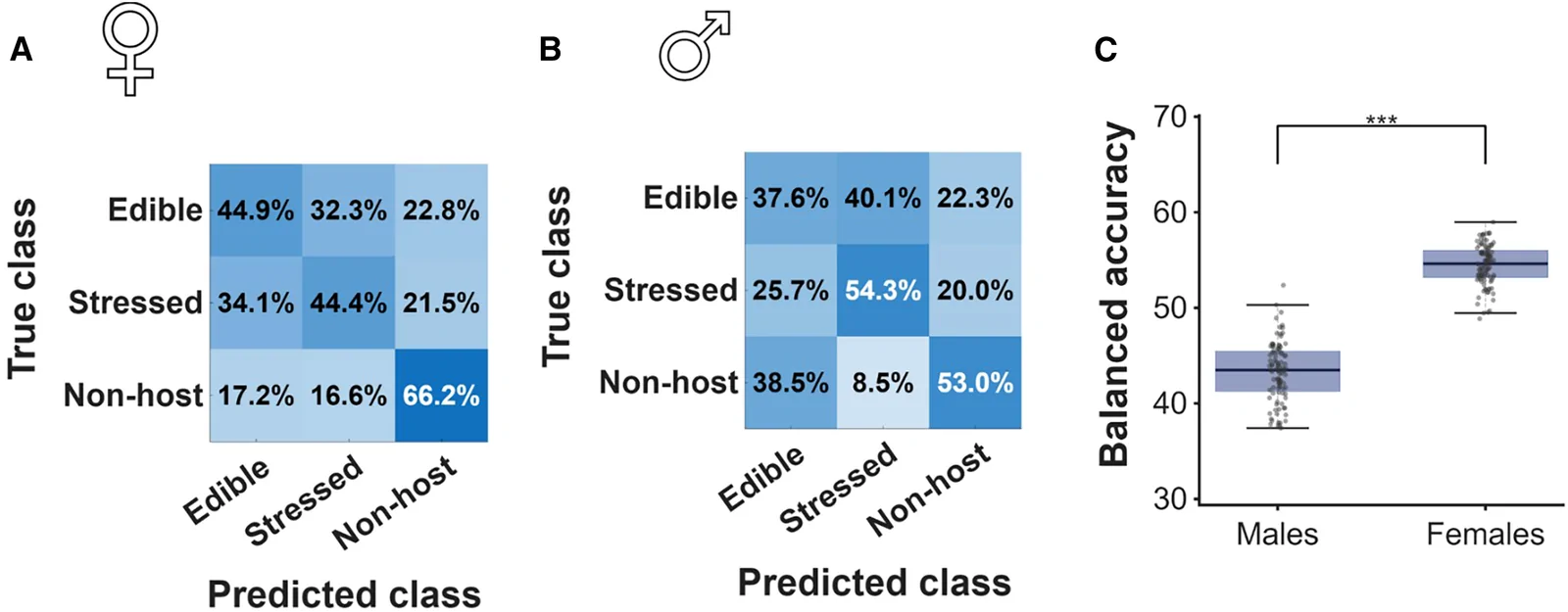
Sex-specific decoding of plant functional categories from antennal electrophysiological signals (A and B) Confusion matrices summarizing classification performance for females (A) and males (B) across functional plant categories (Non-host, Stressed, Host/Edible). Diagonal cells show class-wise recall, whereas off-diagonal cells show the distribution of misclassifications. Balanced accuracy is the unweighted mean of the diagonal recall values across classes. Female decoding accuracy was significantly above chance (54.47 ± 2.12%; *p* = 0.002, permutation test), whereas male performance did not exceed null expectations (43.3 ± 3.1%; *p* = 0.06, permutation test). Values are represented as mean ± SD. Note the male-specific misclassification of non-host stimuli as edible (38.5%) and the shared reciprocal confusion between edible and stressed categories in both sexes. (C) Balanced accuracy per individual across sexes (females: *n* = 54; males: *n* = 36). Females achieved significantly higher overall balanced accuracy than males (*p* = 0.002, GLMM). Boxplots depict the median, interquartile range (IQR), and 1.5 × IQR whiskers; points represent individual mean values. Asterisk indicates statistical significance (*p* < 0.01).

Class-wise performance analyses indicated that higher overall balanced accuracy in females was driven primarily by superior non-host classification. Females exhibited higher recall, precision, and F1-scores for non-host stimuli, whereas males achieved higher performance for the stressed category (both *p* = 0.001, permutation tests; Table S1). Analysis of error distributions further showed that misclassified non-host stimuli in males were predominantly assigned to the edible category (38.5%), a pattern absent in females. However, both sexes exhibited substantial reciprocal confusion between the edible and stressed categories (Figures 3A and 3B).

We then increased task complexity by requiring the model to discriminate among all 12 distinct volatile profiles. Here, both sexes performed significantly above the chance level; however, absolute performance for this fine-grained discrimination remained modest and was likely mostly driven by *N. glauca* and *A. articulata* (balanced accuracies: 25.53 ± 18.56% males; 24.58 ± 20.82% females; permutation test, *p* = 0.001; Figures S3A and S3B). No significant sex differences were detected in the discrimination of individual volatile identities (GLMM, *p* = 0.46; Figure S4). To evaluate potential artifacts introduced by Random Under-sampling (RUS), we repeated the classification analysis on the unbalanced dataset (non-RUS). A comparative analysis confirmed that the primary classification patterns were preserved, indicating that the overall conclusions are robust to class balancing. However, omitting RUS led to a pronounced prediction bias toward specific classes, most notably *Nicotiana glauca*, which was disproportionately predicted across multiple true stimulus categories (Figures S3C and S3D).

### Discrimination of host-plant hydration status

We next asked whether odors provide information about plants’ level of stress. We first compared responses to volatiles from drought-stressed versus well-hydrated tomato plants (*Solanum lycopersicum*). Following established protocols,^37^ drought stress was induced by withholding irrigation for 72 h.

Using the Gradient Boosting classifier, sexual dimorphism was evident again. Female antennal responses successfully distinguished between water-deficit and control volatile profiles, achieving a mean balanced accuracy of 67.31 ± 20.26% (permutation test, *n* = 13, *p* = 0.003; Figure 4A). Conversely, male responses failed to exceed chance expectations, yielding a non-significant mean balanced accuracy of only 33.33 ± 35.36% (permutation test, *n* = 9, *p* = 0.23; Figure 4B; GLMM between sexes, *p* = 0.01, Figure S5A). To control for unequal group sizes, we evaluated classification performance across all 715 unique combinations of nine females selected from the available pool (*n* = 13). The resulting distribution of size-matched female accuracies remained predominantly above the male benchmark, indicating that female performance superiority is robust to sample size constraints (Figure S6).

**Figure 4:**
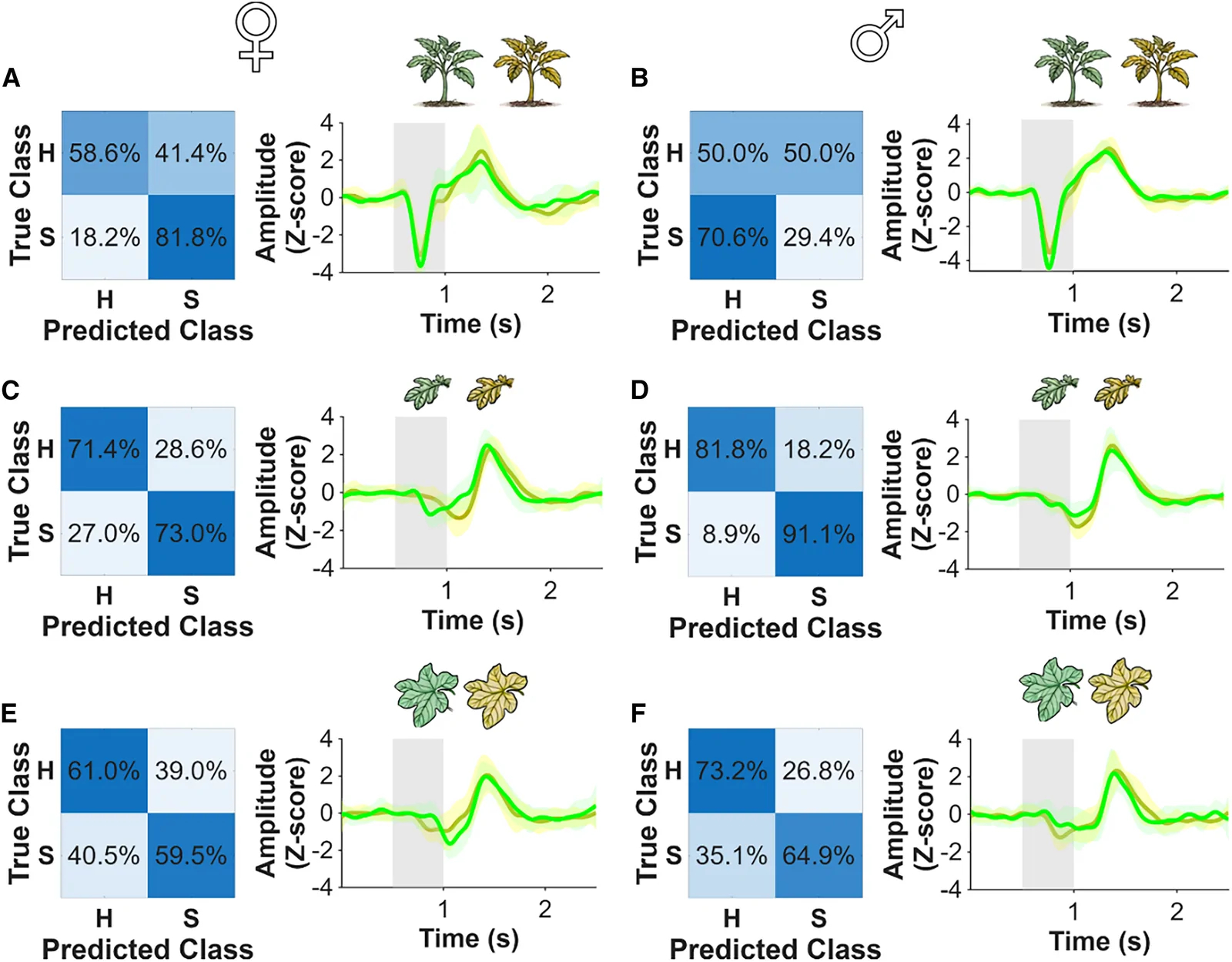
Electroantennographic (EAG) discrimination of host/edible-plant stress status Confusion matrices (left) and corresponding mean temporal antennal responses (right) for females (A, C, and E) and males (B, D, and F) across three experimental conditions. In all matrices, H denotes hydrated controls, and S denotes stressed plants/leaves. Shaded regions around EAG traces represent SD, and gray vertical bars indicate the stimulus delivery period. (A and B) Drought discrimination in tomato (*Solanum lycopersicum*). Matrices illustrate the capacity to distinguish between 72-h drought-stressed (S) and hydrated (H) controls. Females (A) successfully resolved subtle physiological shifts (permutation test, *p* = 0.003, *n* = 13), whereas male performance (B) did not significantly deviate from chance (permutation test, *p* = 0.23, *n* = 9). (C and D) Acute desiccation discrimination in tomato (*S. lycopersicum*). Under extreme stress conditions, both females (C) and males (D) exhibited robust discrimination of extreme physiological divergence (balanced accuracy, mean ± SD; females: 69.79 ± 25.44%, *p* = 0.002, *n* = 16; males: 85.42 ± 14.75%, *p* = 0.001, *n* = 16; GLMM, *p* = 0.19). (E and F) Acute desiccation discrimination in the natal host (*Ricinus communis*). Both sexes exhibited high classification accuracy when distinguishing fresh leaves from those subjected to acute desiccation (Permutation tests; females: *p* = 0.02, *n* = 16; males: *p* = 0.003, *n* = 16; GLMM between sexes, *p* = 0.2).

To further delineate the limits of these discriminatory abilities, we exposed antennae to a high-intensity stress contrast designed to test whether EAG temporal dynamics could distinguish strong physiological divergence in plant tissue. Specifically, we compared responses to freshly excised leaves with responses to excised leaves subjected to acute desiccation (1 h at 30°C and 30% relative humidity; Figures 4C–4F). This treatment was not intended to mimic gradual field drought, but rather to provide an experimentally controlled severe-desiccation condition complementary to the milder 72-h intact-plant water-withholding treatment described above. The comparison was conducted using two plant species: tomato (*S. lycopersicum*), a plant whose volatile profile was not experienced by the adult moth population under our rearing conditions, and castor bean (*R. communis*), the natal host on which the larvae were routinely reared. Because leaf excision and rapid drying may also induce wound-associated volatile emissions and tissue degradation, this treatment should be interpreted cautiously as an acute, high-intensity tissue-stress treatment rather than as drought alone.

In the case of the tomato plants, the physiological divergence between fresh and desiccated tissues elicited highly distinct antennal signals. This resulted in high classification accuracies for both sexes, with no significant difference in performance (females: 69.79 ± 25.44%, permutation test, *p* = 0.002; males: 85.42 ± 14.75%; Figures 4C and 4D; permutation test, *p* = 0.001; GLMM between sexes, *p* = 0.19; Figure S5B). Interestingly, when evaluating the natal host (*R. communis*), the discriminatory signal was less prominent but remained statistically significant for both sexes. Females achieved a mean balanced accuracy of 57.92 ± 20.40% (permutation test, *p* = 0.02; Figure 4E), while males achieved 71.67 ± 27.19% (permutation test, *p* = 0.003; Figure 4F). As with the acute tomato stressor, no significant difference in balanced accuracy was detected between the sexes for the natal host (GLMM, *p* = 0.2; Figure S5C).

To rule out potential artifacts from natural variations in leaf mass or volatile concentration, we evaluated EAG responses to different-sized leaf segments cut from a single well-irrigated tomato plant (*n* = 3, female antennae). Signals were analyzed using the identical processing pipeline. A point-by-point permutation test with max-statistic family-wise error (FWE) correction compared Z-scored EAG trajectories across leaf areas. Response shape and amplitude showed no significant differences at any individual time point (permutation test, minimum *p* = 0.83; Figure S7). These results indicate that normalized trajectories and kinetic profiles remain statistically equivalent regardless of leaf tissue size. These results also align with previous observations using a similar experimental paradigm.^28^

## Discussion

Our results suggest that the temporal dynamics of the electroantennogram (EAG) signal convey ecologically relevant information regarding host-plant toxicity and nutritional suitability, rather than providing a basis for taxonomic identification. The use of intact and stressed plant tissues inherently introduces realistic variability in volatile intensity and composition across individuals and treatments. Rather than treating this variability through a traditional reductionist lens, the analytical framework presented here incorporates it as part of the natural stimulus complexity insects encounter in their environments. That structured differences persist under such a conservative, dynamics-focused framework demonstrates that temporal antennal features may encode information about the odor environment.

Crucially, the observed signal evolution (e.g., Figures 1 and 4) aligns with the insect olfactory system’s established temporal resolution, providing a feasible time frame for the central nervous system to integrate the sequential recruitment of different Odorant Receptor Neuron (ORN) subsets.^38^^,^^39^^,^^40^ This interpretation is consistent with receptor-repertoire studies showing that odor identity, concentration, and stimulus duration can be encoded by distributed and temporally structured activity across olfactory receptors.^2^^,^^41^^,^^42^ For example, receptor-repertoire studies in *Drosophila* show that broadly and narrowly tuned receptors, and their excitatory and inhibitory response profiles, can jointly define a multidimensional odor space.^2^ Although EAG cannot resolve individual receptor or OSN activity, such receptor-level dynamics provide a plausible basis for the integrated temporal antennal profiles measured here.

During sample collection and confinement, plants emit Green Leaf Volatiles (GLVs), primarily alcohols, aldehydes, and esters, which are common across diverse taxa. The similarity of GLV profiles among species makes it difficult to distinguish individual plants or group them by evolutionary relationships based on these volatiles.^43^ For polyphagous herbivores, plant relatedness is a poor proxy for fitness-relevant traits: closely related species can vary in suitability and defenses, while convergent chemical traits can arise independently across distant taxa.^44^

Based on our limited survey here, the neural representation of odor space appears to be organized primarily by the ecological significance of volatiles rather than their phylogenetic origins. Our data imply that when volatiles are grouped by chemical function (e.g., edible, stressed, non-host), the differentiation within the odor space becomes significantly more pronounced (Figure 3 and Table S1). Specifically, EAG responses may reveal a sensory system tuned to functional affordances: the survival or reproductive opportunities a plant provides, rather than taxonomic identity. These results are consistent with the early observations of Prasad and Bhattacharya,^30^ who examined the growth and survival of *S. littoralis* across a diverse range of host plants (including *R. communis*, *C. album*, and *S. lycopersicum*). Their findings revealed no relationship between larval performance and the phylogenetic relatedness of the host plants tested.

Our results similarly highlight a fundamental mismatch between insect-relevant functional responses and plant taxonomy, a pattern that may be explained by the continuous structure of the olfactory landscape. By representing functional similarity as continuous local gradients rather than discrete clusters, the odor space offers a neurobiological framework for understanding why taxonomically diverse host plants can evoke overlapping response patterns.

Notably, although classification performance exceeded chance levels, absolute accuracies remained moderate. Nevertheless, this sensory tuning maintains a boundary for non-host taxa, which occupy distinct peripheral regions of the odor space in both sexes. This spatial segregation by non-hosts persists during supervised classification. However, although *A. articulata*, *N. glauca*, and *S. elaeagnifolium* cluster closely, they lack internal differentiation and are consistently grouped within a shared peripheral “non-host” zone rather than forming discrete subclusters (Figure S2). Class-wise analyses further showed that this pattern was particularly pronounced in females, which exhibited higher recall, precision, and F1-score for the non-host category than males (Table S1).

Within this graded sensory landscape, we suggest a sex-specific asymmetry in the distribution of responses. At the behavioral level, such higher-resolution peripheral filtering may bias females toward further inspection of host plants, including approach, landing, probing, and eventual oviposition acceptance. In the mild tomato drought task, females outperformed males (67.3% vs. 33.3% balanced accuracy; *p* = 0.01), with only female performance exceeding chance, consistent with finer female discrimination under subtle stress conditions (Figure 4). These results contrast, to some extent, with the higher classification performance of males for stressed plants (Table S1), but may also indicate that antennal responses provide context-dependent information for downstream processing rather than independently determining oviposition-site selection.

This observation extends earlier findings by Anderson et al.^32^ While they observed similar response magnitudes across sexes, our analysis of the temporal signal profile reveals a potential sexual dimorphism. We hypothesize that this sexual dimorphism might reflect divergent ecological pressures: while male olfaction is tuned for foraging and pheromone tracking, females require higher sensory resolution to navigate botanical complexity and optimize host selection for larval survival. These results suggest that temporal response dynamics provide an additional dimension of antennal signaling that complements conventional measures of response amplitude. Whether these peripheral differences influence approach, probing, or oviposition decisions remains to be determined.

This does not imply male insensitivity to plant volatiles. Rather, it indicates that, at least within the botanical taxa sampled in this study, male peripheral dynamics support functional categorization with less consistency than females, particularly during fine discrimination tasks. Notably, in many insect species, males use GLVs as essential olfactory cues for locating females. This phenomenon is also well exemplified in virgin *S. littoralis* males, which exhibit high sensitivity and strong attraction to cotton-derived volatiles.^45^^,^^46^ Consistent with this, our experiments show that under high-contrast ecological conditions, male antennae retain the capacity to discriminate between healthy and stressed hosts’ GLVs (Figure 4). For males, this coarser organization may nevertheless be adaptive if plant volatiles are used primarily for rapid orientation toward feeding sites or habitats where females are likely to occur, rather than for fine-scale evaluation of larval host quality. This may have implications for attraction behavior and foraging decisions in heterogeneous environments, including conditions in which severe drought stress alters nectar quality.^47^ In an earlier behavioral experiment, we found that female moths preferentially located and oviposited on hydrated rather than dehydrated plants^37^ (Figure S8). These findings further support both the capacity and the ecological relevance of female discrimination between these two plant states.

Interestingly, both sexes exhibited a significantly higher capacity to discriminate between fresh and acutely desiccated tomato leaves than between the corresponding castor bean treatments (Figure 4). Within the tomato comparison, males showed numerically higher classification accuracy than females (85.42% vs. 69.79%), although this difference was not statistically significant (*p* = 0.19). This pattern may reflect sex-specific sensitivity to the novel, high-intensity volatile blend or differences in signal quality between sexes, but these explanations remain speculative, and larger datasets alongside behavioral experiments will be required to evaluate these possibilities. More broadly, these findings suggest that larval development on a specific host plant does not necessarily confer a subsequent advantage in assessing that host’s physiological state as an adult, although long-term laboratory rearing may also have reduced experience-dependent plasticity. Nevertheless, the results should be interpreted with caution. The larvae in our study were reared under controlled laboratory conditions with a constant supply of high-quality foliage, which may differ from the resource variability encountered in the field. In addition, as was discussed above, this inference is limited to peripheral antennal responses and does not directly predict adult foraging or oviposition decisions.

While EAG measures peripheral sensitivity, these physiological signals represent only the initial stage of the complex, multimodal behavioral framework governing host selection in *S. littoralis*. In this species, foraging and oviposition are protracted processes characterized by a sequential transition from long-range olfaction to short-range contact chemoreception and probing.^6^^,^^48^

Within this hierarchy, EAG-derived features should be interpreted as peripheral inputs that may bias or prioritize subsequent processing, rather than as direct readouts of behavioral choice. Importantly, these results should not be interpreted as evidence that the antenna alone performs organization according to biologically defined plant groups or determines behavioral decisions. The antennal signal is likely transformed in the antennal lobe and higher brain centers, where temporal input patterns can be integrated, sharpened, suppressed, or combined with other sensory and internal-state information.^49^ Thus, the antenna may provide the first stage of sensory processing, encoding amplitude and temporal dynamics for downstream neural circuits, while behavioral decisions emerge from subsequent neural processing.^50^^,^^51^^,^^52^

Consequently, the sex-specific olfactory dynamics observed in the present study do not imply an exclusive reliance on antennal input; rather, they highlight the role of olfaction as a potential precursor within an integrative strategy that incorporates visual, mechanosensory, and acoustic cues.^37^^,^^53^^,^^54^^,^^55^^,^^56^^,^^57^^,^^58^ Thus, future work linking antenna-derived temporal features to extended behavioral sequences, such as approach behavior, probing duration, and acceptance likelihood, will be essential, particularly when incorporating contact-based input and other modalities within a unified analytical framework.

An important limitation of the present study is that the volatile blends were not characterized using gas chromatography-mass spectrometry/electroantennographic detection (GC-MS/EAD) or an equivalent chemical-profiling approach. Consequently, we cannot identify the compounds, blend ratios, emission levels, or treatment-associated volatiles underlying the observed temporal EAG differences. Moreover, because natural plant odors vary intrinsically in composition and concentration, stimuli could not be fully standardized across plant species and physiological states. Although such variability is an inherent feature of the natural olfactory environments encountered by insects,^4^ our results should not be interpreted as demonstrating ecological organization independently of chemical variation. Rather, they show that integrated peripheral responses to natural odor blends contain structured information associated with the biologically defined plant groups examined here. Temporal EAG analysis and GC-MS/EAD should therefore be viewed as complementary: chemical profiling can characterize blend composition and identify electrophysiologically active compounds, whereas temporal EAG preserves the integrated antennal response to the complete volatile blend.^23^^,^^24^

Although some classification analyses were based on relatively small numbers of individuals, the main conclusions were supported by leave-one-individual-out cross-validation, permutation testing, and sample-size-matched sensitivity analyses designed to reduce potential sample-size-related bias; nevertheless, replication in larger independent datasets will allow more precise estimation of classification performance and individual variability.

In summary, our results support a model in which the peripheral olfactory system of a polyphagous moth organizes complex plant odor signals along biologically meaningful dimensions. Female moths, in particular, exhibit more coherent functional organization of these signals, especially under fine-grained discriminations that are likely to be decision-relevant. This supports the broader life-history hypothesis underlying the study: sensory systems need not identify a plant in taxonomic terms, but rather extract information about its potential fitness relevance, thereby enabling adaptive decision-making in chemically diverse and dynamic environments.

More broadly, the approach developed here provides a framework for studying olfactory ecology beyond *S. littoralis*. By preserving the complexity of natural odor scenes while quantifying how insects physiologically organize them, this methodology may help identify candidate cues for subsequent behavioral assays in pests and pollinators.

### Limitations of the study

The strengths of this study include the use of natural plant odor blends and complementary analytical approaches to characterize temporal EAG dynamics under ecologically relevant conditions. However, several limitations warrant discussion and also highlight important directions for future research. While the absence of GC-MS/EAD limits direct comparison between the physicochemical space of the odor blends and the EAG-derived response space, such comparisons represent an important avenue for future work because physical similarity does not necessarily correspond to similarity in the receiver’s perceptual space.^59^ In addition, natural plant odors could not be fully standardized across species and physiological states. EAG reflects summed peripheral antennal activity and does not resolve individual receptor contributions or subsequent central processing, so these responses should not be interpreted as direct predictors of behavior. Finally, the relationship between antennal response dynamics and behavioral responses has not been studied and should be examined in future work.

## Resource availability

### Lead contact

Further information and requests for resources should be directed to Yossi Yovel (yossiyovel@gmail.com).

### Materials availability

This study did not generate new unique reagents or other new materials.

### Data and code availability

#### Data

The electroantennography signals table and associated datasets generated during this study have been deposited at Mendeley Data and are publicly available as of the date of publication. Accession links and DOIs are listed in the key resources table.

#### Code

All original code, scripts, and functions (MATLAB) generated during this study have been deposited at Mendeley Data and are publicly available as of the date of publication. Accession links and DOIs are listed in the key resources table.

#### Any additional information

Any additional information required to reanalyze the data reported in this paper (including hardware modifications such as the 3D-printed antenna holder) is available from the lead contact upon request.

## Acknowledgments

We thank Prof. Amir Ayali for allowing us to use his experimental system.

We thank Dr. Rya Seltzer and Dr. Danny Minahan for reviewing the manuscript.

## Author contributions

Conceptualization, G.Z.E., N.S., and Y.Y.; methodology, G.Z.E. and N.S.; investigation, G.Z.E. and N.S.; data curation, G.Z.E. and N.S.; software, G.Z.E. and N.S.; formal analysis, G.Z.E. and N.S.; visualization, G.Z.E., N.S; resources, G.Z.E., M.D., and D.M.; writing – original draft, G.Z.E.; writing - review and editing, G.Z.E., N.S., and Y.Y.; project administration, G.Z.E. and Y.Y.; supervision, Y.Y.; funding acquisition, Y.Y.

## Declaration of interests

The authors declare no competing interests.

## Declaration of generative AI and AI-assisted technologies in the manuscript preparation process

During the preparation of this work the authors used Gemini pro in order to review and edit the codes and the manuscript. This tool was used solely for language polishing and code formatting and not in any way to directly analyze the data or write the current text. After using this tool/service, the authors reviewed and edited the content as needed and take full responsibility for the content of the published article.

## STAR★Methods

### Key resources table

**Table:** 

REAGENT or RESOURCE	SOURCE	IDENTIFIER
**Experimental models: Organisms/strains**		

*Spodoptera littoralis*	Volcani Institute	N/A
*Solanum lycopersicum*	Growth-room culture	N/A
*Ricinus communis*	Tel Aviv Zoological Garden	N/A
*Nicotiana glauca*	Tel Aviv Zoological Garden	N/A
*Solanum elaeagnifolium*	Tel Aviv Zoological Garden	N/A
*Anabasis articulata*	Tel Aviv Zoological Garden	N/A
*Chenopodium album*	Tel Aviv Zoological Garden	N/A
*Capsicum* sp.	Tel Aviv Zoological Garden	N/A

**Software and algorithms**		

Python	Python Software Foundation	v3.9.19
MATLAB	MathWorks	R2025b
phyloT	phyloT	https://phylot.biobyte.de
Signals table and raw datasets	This paper	https://data.mendeley.com/datasets/kbxmpxpmkd/1
Analysis code	This paper	https://data.mendeley.com/datasets/kbxmpxpmkd/1

**Other**		

IDAC-2 data acquisition interface	Syntech	IDAC-2
CS-55 stimulus controller	Syntech	CS-55
Digital Temperature and Humidity Data Logger	Elitech	GSP-5G
Modified 3D-printed antenna holder	Neta et al.^28^	N/A

### Experimental model and study participant details

#### Spodoptera littoralis

*Spodoptera littoralis* larvae were reared on castor bean (*R. communis*) leaves at the Volcani Institute under controlled conditions (25 ± 1 °C, 40% RH, and a 12:12 h L:D photoperiod). Insects were transported as pupae to Tel Aviv University and maintained under identical environmental parameters. To ensure the use of virgins, newly emerged adults were sex-separated daily. Adults were housed in 2 L boxes and provided with a 60% (w/v) sucrose solution *ad libitum*.

#### Plants

For the mildly stressed plant experiments, young tomato plants (*Solanum lycopersicum*) were grown in a growth room at 25 °C and kept in long-day conditions (16 h day, 8 h night). Once plants reached a medium size, irrigation was withheld for three consecutive days to induce mild drought stress. Control plants were selected from the same group of plants and maintained adjacent to the stressed plants, receiving daily irrigation.

For the severely stressed plant experiments, wild tomato (*S. lycopersicum*) and castor bean (*Ricinus communis*) plants were collected from the Tel Aviv Zoological garden. To induce severe stress, leaves were excised from the plants and subjected to controlled drying for 1 h at 30°C and 30% relative humidity. Control leaves from the same individual plants were excised immediately prior to the start of the experiment. Similarly, for all other measurements involving wild plants, leaves and flowers were collected adjacent to the experimental setup and replaced every 1–1.5 h to ensure stimulus consistency. This included three alkaloid-rich species toxic to larval development (*Nicotiana glauca* [leaves and flowers assayed separately], *Solanum elaeagnifolium*, and *Anabasis articulata*) and two more species known to support larval growth (*Chenopodium album*, and *Capsicum* sp.).

### Method details

#### The electroantennogram recordings

Electroantennogram (EAG) recordings were conducted on 2–4 days old adult moths of both sexes. Prior to antennal excision, individuals were anesthetized by exposure to-20 °C for 3–5 min. Both antennae were then excised at the base, proximal to the pedicel, using micro-scissors, and were mounted on a custom-made antenna holders for recording. The 3D-printed antenna holder described in Neta et al.^28^ (Raise Pro2, 3D Printer) was modified to fit moth antenna dimensions. EAG responses were recorded following the protocol described by Neta et al.^28^ using the same recording system. Signals were amplified (×100), band-pass filtered (1 Hz high-pass; 500 Hz low-pass), and digitized at 100 Hz via an IDAC-2 interface using EAGPro software (Syntech).

To assess EAG responses to leaf volatiles, several leaves of comparable size were excised from each treatment group and placed inside a 10 mL plastic tube perforated at both ends. The tube was connected on one end to a stimulus controller (CS-55, Syntech, Germany) and on the other to a custom-built Y-shaped tube, enabling simultaneous stimulation and recording from both antennae, thereby improving internal consistency and experimental throughput.

Each stimulus was presented in three repetitions at 1-min intervals. Empty tubes were used as controls to establish baseline antennal responses to the carrier air stream.

#### Raw data post-processing

A total of 90 individual moths were utilized (54 females, 36 males), yielding 1,072 signals with a resolution of 250 samples per signal (2.50 s at a sampling rate of 100 Hz). Same sample indices were used for every trial. No trace-specific truncation, temporal pre-alignment, resampling, or zero-padding was applied.

Electroantennogram (EAG) signals were processed using custom Python (Python 3.9.19, Software Foundation, Scotts Valley, CA) and MATLAB (MathWorks, R2025b) pipelines. To isolate odor-evoked activity, the average response to three blank control trials was subtracted from the experimental traces of the same antenna.

To remove high-frequency noise while preserving the physiological features of the EAG signal, a third-order Butterworth low-pass filter with a 50 Hz cutoff was applied. Trials exhibiting low signal-to-noise ratio (SNR) were excluded from the analysis. To emphasize temporal dynamics over absolute amplitude, each post-stimulus trace was *Z* score normalized.

*Z* score normalization was applied before derivative dynamic time warping (DDTW) to reduce the influence of baseline offsets and absolute amplitude differences among antennal preparations, allowing similarity comparisons to focus on normalized waveform morphology. This preprocessing is appropriate for time-series analyses in which the aim is to compare structural similarity while minimizing amplitude- and offset-driven distortions. It is particularly relevant for DDTW because the distance calculation is based on local derivative structure rather than raw signal magnitude; after normalization, these derivatives represent relative depolarization and recovery dynamics in standardized units. Thus, the combined use of *Z* score normalization and DDTW emphasizes temporal response shape while preserving real differences in the timing and sequence of waveform features.^60^^,^^61^ Z-score-normalized EAG traces were first smoothed using a third-order Savitzky-Golay filter with an 11-sample window. Local temporal derivatives were then estimated using the Keogh-Pazzani derivative formulation, with the first and last derivative values assigned from the nearest internal estimates. DTW was applied to the derivative sequences using Euclidean local distances, with no warping-window constraint. No temporal downsampling was applied.

#### Stimulus-delivery standardization and leaf-load control

To minimize variation in the physical properties of stimulus delivery, all recordings were conducted using the same standardized stimulation system. Air pressure, airflow velocity, temperature, humidity, tubing configuration, stimulus-tube volume, pulse duration, and interstimulus interval were kept constant across treatments. Relative humidity at the stimulus outlet was independently verified using an Elitech GSP-5G Digital Temperature and Humidity Data Logger. Humidity was 68% both with an empty stimulus tube and with a large tomato leaf load tested (3.5 cm^2^), indicating that the presence of leaf material did not measurably alter humidity under the experimental conditions.

To evaluate whether variation in plant-material amount influenced EAG amplitude or temporal response properties, an additional leaf-load control was performed using fresh, well irrigated tomato (*S. lycopersicum*) leaves of three standardized areas: 0.6, 2.8, and 3.5 cm^2^. Each leaf-area condition was tested three times on three female antennal preparations, with the order of presentation varied among preparations. Responses were processed using the same pipeline applied to the main dataset. Maximum and minimum response amplitudes were quantified from the raw signals, while peak latency was calculated from both raw and z-score-normalized traces. Normalized temporal response profiles were additionally compared across leaf-area treatments.

A point-by-point non-parametric permutation analysis was performed and a Kruskal-Wallis test was conducted to evaluate the effect of leaf area on the signal amplitude at each individual time point. To correct for multiple comparisons across the 250 time-steps and control the Family-Wise Error (FWE) rate, we employed the single-threshold max-statistic permutation method. A null distribution was constructed by randomly shuffling the treatment labels 1,000 times across all time points. The corrected *p*-value at each time point was determined as the proportion of shuffles where the permuted maximum statistic exceeded the observed statistic (Figure S6).

### Quantification and statistical analysis

#### Hierarchical clustering and comparison of EAG-derived odor spaces

To assess similarities in EAG responses, we performed agglomerative hierarchical clustering using the average linkage method, stratified by sex, to explore potential differences in sensory organization between males and females.

Cluster stability was evaluated via bootstrap resampling (1,000 iterations) across individual moths. For each sex, a consensus dendrogram was generated, and the median cophenetic correlation coefficient was computed to assess the internal consistency of the resulting hierarchical structures. To statistically compare male and female clustering topologies, we conducted a local permutation test (1,000 permutations) on the representative distance matrices (RDMs), identifying odorants where sex-based sensory divergence was most evident.

To compare female and male odor-response spaces directly, analyses were restricted to odor groups represented in both sexes. Following alignment of the common odor groups, sex-specific distance matrices, hierarchical trees, and two-dimensional non-metric multidimensional scaling (nMDS) representations were recalculated. nMDS was performed using Kruskal stress, with up to 5,000 optimization iterations and 40 random initializations; the solution with the lowest stress value was retained. The nMDS representations were used for visualization of the consensus distance structure.

Physiological discriminability of ecological odor categories (e.g., Non-host, Host/Edible, Stressed) was quantified by comparing the Empirical Cumulative Distribution Functions (ECDFs) of within-versus between-category pairwise distances.

Agreement between male and female cluster partitions was assessed using the Adjusted Rand Index (ARI) across multiple dendrogram cut heights. Additionally, the local structure of the sensory space was compared via the Jaccard similarity of k-nearest-neighbors. Statistical significance of these metrics was evaluated by comparing the observed Area Under the Curve (AUC) across all resolutions to a null distribution generated from 1,000 random label permutations. This allowed us to determine whether the organization of the olfactory space is fundamentally sexually dimorphic (Figure S1).

To explore whether moth sensory space reflects the phylogenetic relationships of plants, we compared the EAG derived physiological distance matrices to a plant phylogenetic distance matrix. The phylogeny was reconstructed using phyloT (based on NCBI taxonomy), and pairwise distances were extracted from the resulting Newick tree.

Correspondence between EAG response similarity and plant phylogenetic relatedness was quantified using a Mantel test with Spearman’s rank correlation. Permutation testing (1,000 permutations) was performed separately for male and female datasets to determine whether evolutionary signal detection is conserved across sexes.

#### Supervised classification

To quantify physiological separability between antennal responses to various volatiles, we implemented supervised classification using Gradient-Boosted decision tree ensembles. The models were trained on the *Z* score normalized signal vectors, using temporal response kinetics as the primary input feature. Before classification, signals were low-pass filtered using a fifth-order Butterworth filter with a 50-Hz cutoff and independently *Z* score normalized. Signal quality was evaluated using a composite quality-control score incorporating the signal-to-noise ratio, the post-stimulus to baseline energy ratio, peak response amplitude, and baseline variability. The lowest 15% of responses according to this score were excluded from further analysis. The signal-to-noise ratio was calculated as:

$${\text{SNR}}_{dB}=20\;\log_{10}\left(\frac{{\text{RMS}}_{post}+\varepsilon }{{\text{RMS}}_{baseline}+\varepsilon }\right)$$

where RMS_*baseline*_ and RMS_*post*_ represent the root-mean-square signal amplitudes during the baseline (pre-stimulus) and post-stimulus periods, respectively.

To maintain biological validity and prevent data leakage, we used group-aware partitioning, ensuring that all repetitions from a single moth were exclusively assigned to either the training or test set.

Model generalization was further assessed using leave-one-individual-out cross-validation across all groups. These approaches allowed classification performance to reflect population-level sensory patterns rather than individual-specific idiosyncrasies. To address class imbalances among plant species and treatments (Figures S2A and S2B), Random Under-sampling (RUS) was applied during the training phase. The held-out test data were not undersampled. As a sensitivity analysis, the complete classification procedure was repeated without Random Under-sampling, thereby retaining all available training observations (Figures S2C and S2D).

The classifier used 800 boosting iterations, a learning rate of 0.001, and decision-tree learners with a maximum of 16 splits and a minimum leaf size of eight observations. Uniform class priors were applied. For multiclass classification, GentleBoost learners were combined using a one-versus-all error-correcting output-code framework. A fixed random seed was used to ensure reproducibility. Hyperparameters were fixed before analysis and were not optimized using the test data.

To evaluate whether superior female classification performance was driven by sample size disparities, we repeated the analysis using unique, size-matched subsets of females drawn from the whole group. Each subset was evaluated using the identical Gradient-Boosting classifier and leave-one-individual-out cross-validation framework applied in the primary analysis. These results confirm that higher female decoding accuracy was not an artifact of unequal sample sizes (Figures 3 and S5 and Table S1).

Classifier performance was evaluated using balanced accuracy, a robust metric that accounts for disparities across categories. Results were visualized using confusion matrices normalized by true class counts. For each sex, the statistical significance of classification performance was determined through permutation testing (1,000 iterations), generating null distributions by randomly shuffling class labels.

This supervised approach was conducted at two levels: (1) discrimination among individual plant species, and (2) classification into functional ecological categories: Non-host, Stressed, or Host/Edible.

Sex differences in classification performance were evaluated using a generalized linear mixed-effects model with a binomial error distribution and logit link. Classification outcome for each held-out observation was coded as correct (1) or incorrect (0). Sex was included as a fixed effect, while moth identity was included as a random intercept to account for repeated observations from the same individual. Stimulus class was included as an additional fixed-effect Model convergence and singularity were examined from optimizer diagnostics and random-effect estimates.

### Additional resources

This study did not generate additional resources.
