# Supplementary material for: Temporal dynamics of antennal responses encode plant stress and ecological relevance in Spodoptera littoralis moths
Source: iScience. 2026 Oct 22;29(11):117941. doi: 10.1016/j.isci.2026.117941 (PMC13718390; doi:10.1016/j.isci.2026.117941)
Supplement: Supplementary file 1 [file mmc1.pdf]

iScience, Volume *29*

Supplemental information

Temporal dynamics of antennal responses encode plant stress and ecological relevance in *Spodoptera littoralis* moths

Guy Zer Eshel, Neta Shvil, Michael Davidovitz, Dana Ment, and Yossi Yovel

Supplementary materials

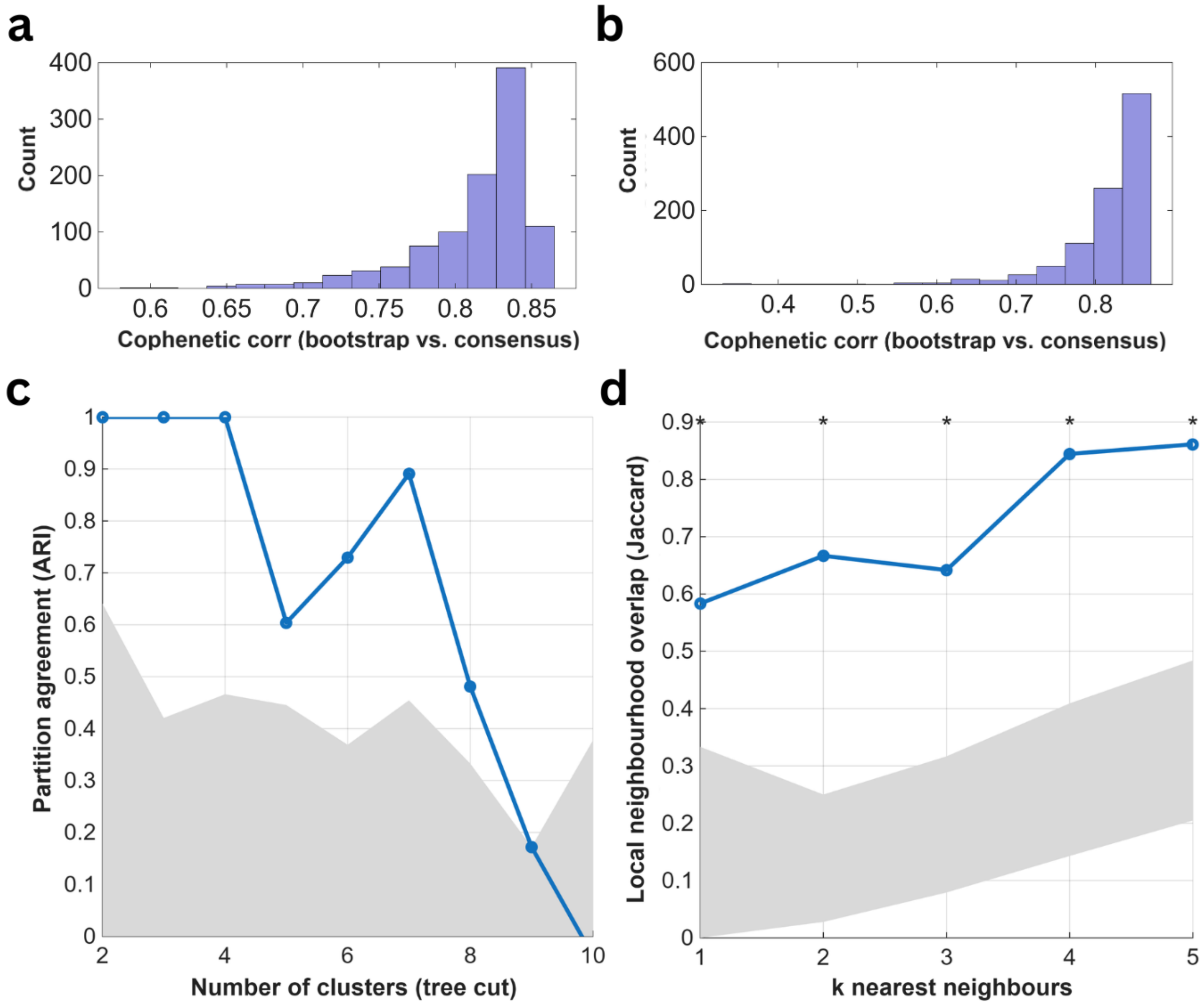

Figure S1. Robustness and statistical validation of unsupervised hierarchical clustering of electroantennogram (EAG) responses. (a, b) Structural stability of odor representations for females (n = 54) and males (n = 36). Histograms show the distribution of cophenetic correlation coefficients between 1,000 bootstrap-resampled iterations and the consensus dendrogram. High median correlations (Females: r = 0.87; Males: r = 0.83) indicate a stable hierarchical organization resilient to individual variance. **(c)** Partition agreement across dendrogram cut heights (2-10 clusters). The blue line represents the observed Adjusted Rand Index (ARI); the gray shaded area denotes the 95% confidence interval of the null distribution (1,000 permutations). **(d)** Local neighborhood stability assessed via Jaccard similarity of k-nearest neighbors (k = 1 to 5). Asterisks denote significant deviations from the null distribution ($p < 0.001$, permutation test), confirming that the EAG-based odor space is non-random and biologically structured.

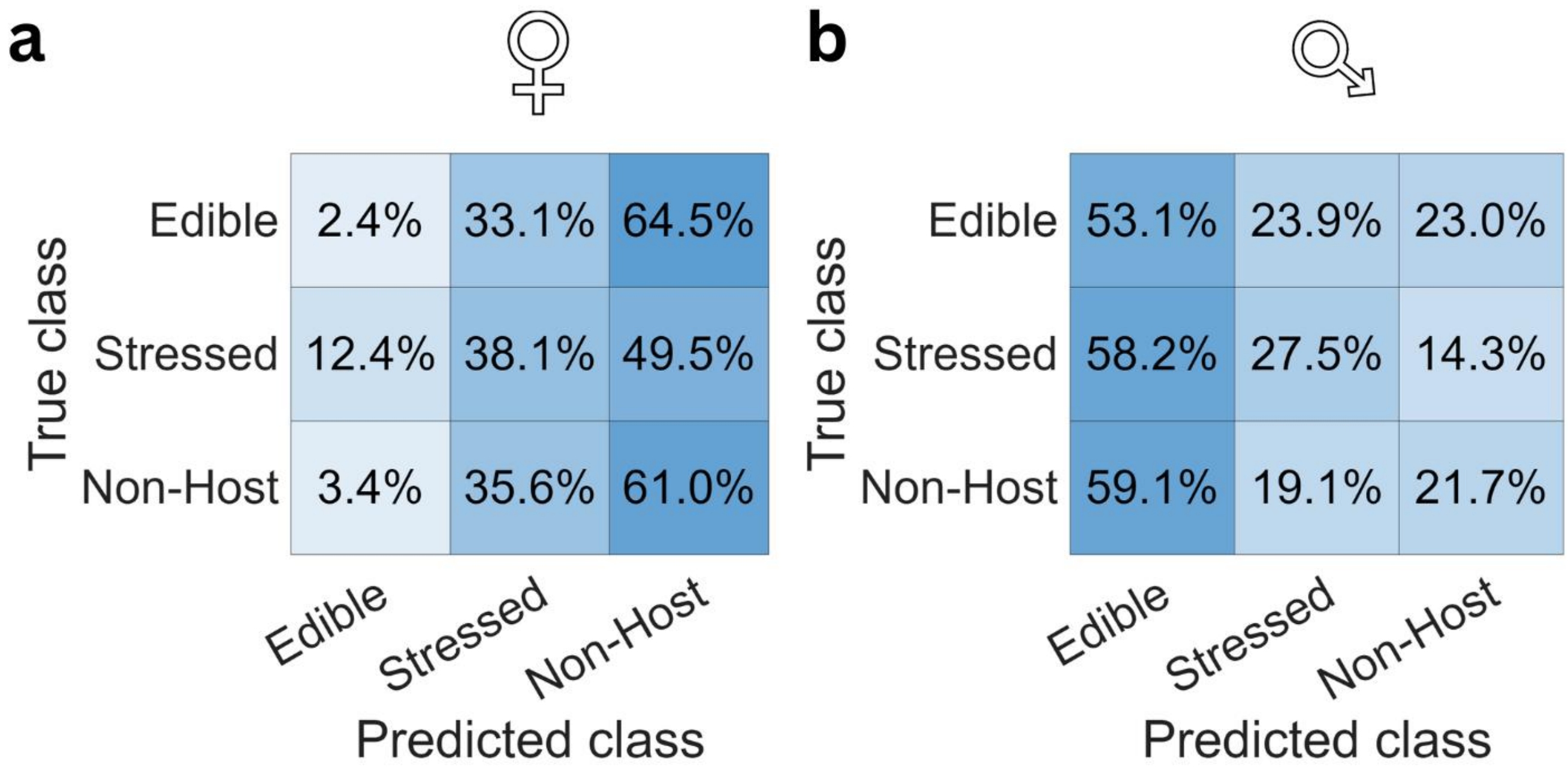

Figure S2. Classification performance using area under the curve (AUC) as the sole predictor. Row-normalized confusion matrices show the performance of AUC-based Gaussian naive Bayes classifiers for **(a) females** and **(b) males**. Classifiers were evaluated using leave-one-moth-out cross-validation, with Random Undersampling applied only to the training data to balance ecological classes. Rows indicate the true class and columns the predicted class; values represent the percentage of observations within each true class assigned to each predicted class.

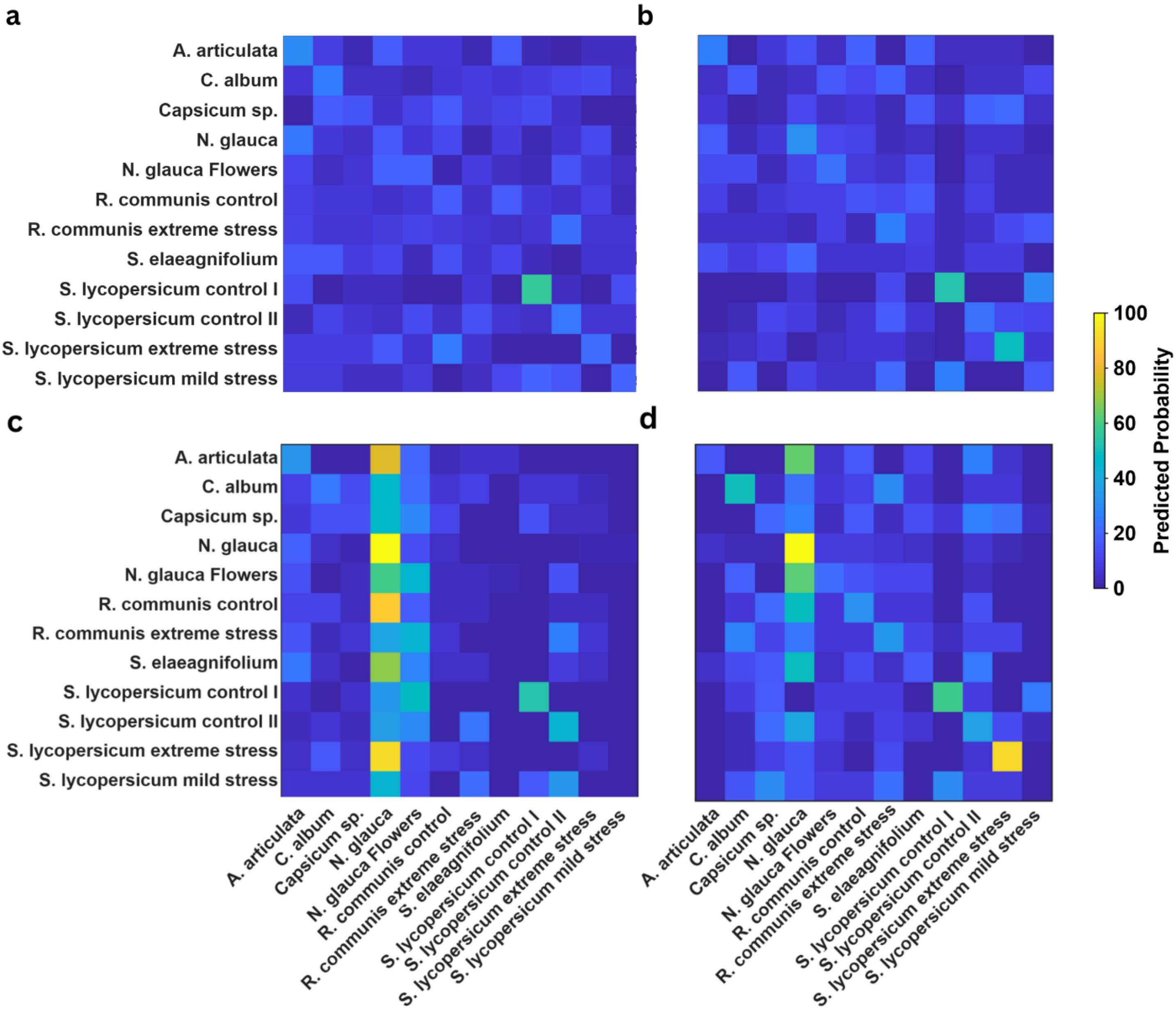

Figure S3. Confusion matrices for Gradient Boosting discrimination of 12 distinct volatile profiles. Classification performance for **(a)** females and **(b)** males across all individual plant-odor identities. Each cell represents the percentage of instances where an odor from plant (y-axis) was classified as plant (x-axis) by the model. The diagonal indicates correct classifications (balanced accuracy), while off-diagonal elements illustrate specific patterns of perceptual overlap or misclassification between volatile profiles. Both sexes performed significantly above the chance level (permutation test, $p < 0.001$). Note the high degree of similarity between certain *Solanum lycopersicum* treatments, which may account for the majority of classification errors. Groups *S. lycopersicum* control I and control II serve as the respective baselines for the extreme and mild stress treatments. Panels (**c**) (females) and (**d**) (males) show the corresponding results without RUS. Comparison of the RUS and non-RUS analyses shows that the main classification patterns are retained even when all available samples are included, indicating that the overall conclusions are not dependent on the use of random undersampling. The color bar indicates the percentage of classification instances, ranging from 0% (purple) to 100% (yellow).

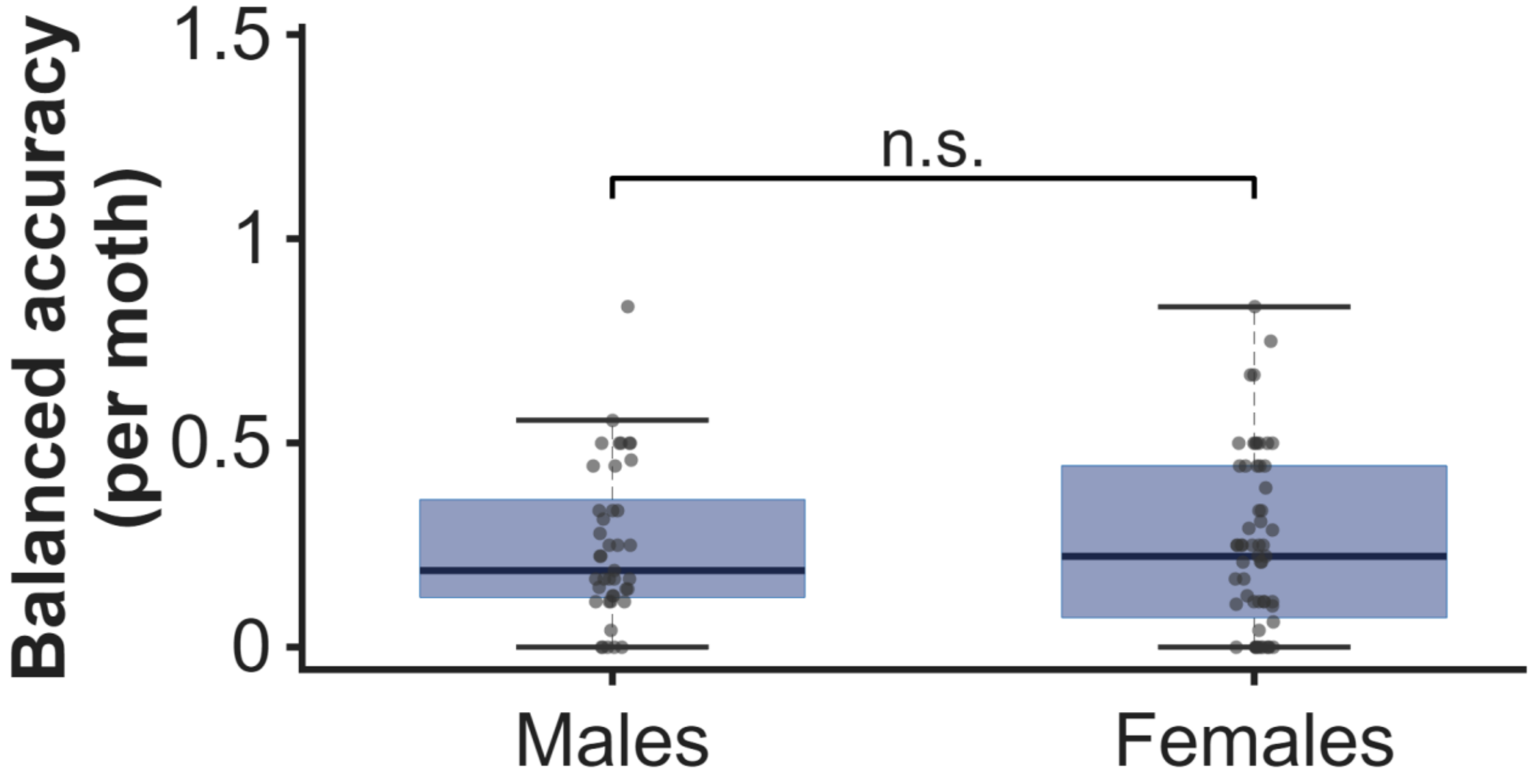

Figure S4. Sex-specific classification performance across plant species and functional categories. Balanced accuracy metrics for male and female *Spodoptera littoralis* peripheral responses, calculated using a Gradient-Boosting classifier with a "Leave One Individual Out" cross-validation framework. Comparative balanced accuracy per individual moth (dots) for the 12 distinct volatile profiles. While both sexes significantly exceeded chance (permutation test, $p = 0.001$), no significant difference was found between male (25.53 ± 18.56%; mean ± SD) and female (24.58 ± 20.82%; mean ± SD) performance (GLMM, $p = 0.46$). Horizontal lines represent the median, with boxes spanning the interquartile range (IQR) and whiskers extending to 1.5× IQR. "n.s." indicates no significant difference between sexes.

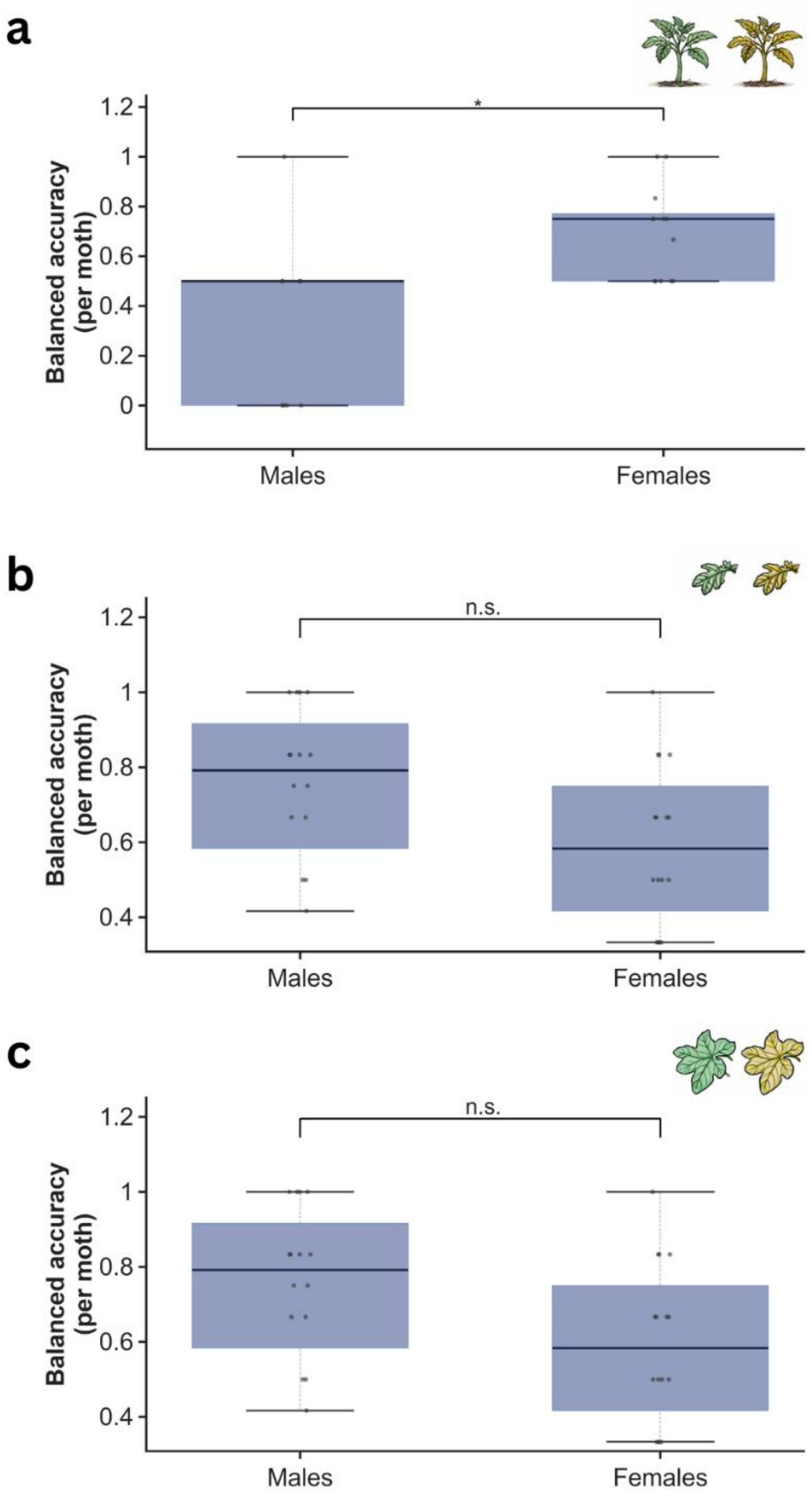

Figure S5. Sex-specific classification of plant physiological states based on antennal odor representation. Balanced accuracy (per moth) for male and female *S. littoralis* antennal responses, computed using a Gradient Boosting classifier. (**a**) Drought stress vs. well-watered *S. lycopersicum*: Females (n = 13) exhibited significantly higher classification performance compared to males (n = 9) (GLMM, p = 0.01). (**b**) Fresh vs. acutely desiccated *S. lycopersicum* leaves: No significant sex-dependent differences in classification accuracy were observed (n = 16 per sex; GLMM, p = 0.19). (**c**) Fresh vs. acutely desiccated *R. communis*: Performance was comparable between sexes (n = 16 per sex; GLMM, p = 0.2). Horizontal lines indicate medians; boxes represent interquartile ranges (IQR); whiskers extend to 1.5× IQR. Individual points correspond to single moths. On the upper-right corner of each plot: whole plants show mildly stressed (yellow) vs. control (green) tomato leaves; extremely stressed (yellow) vs. control (green) tomato leaves; stressed (yellow) vs. control (green) castor bean leaves. Statistical significance is indicated as $p < 0.05$; n.s., not significant.

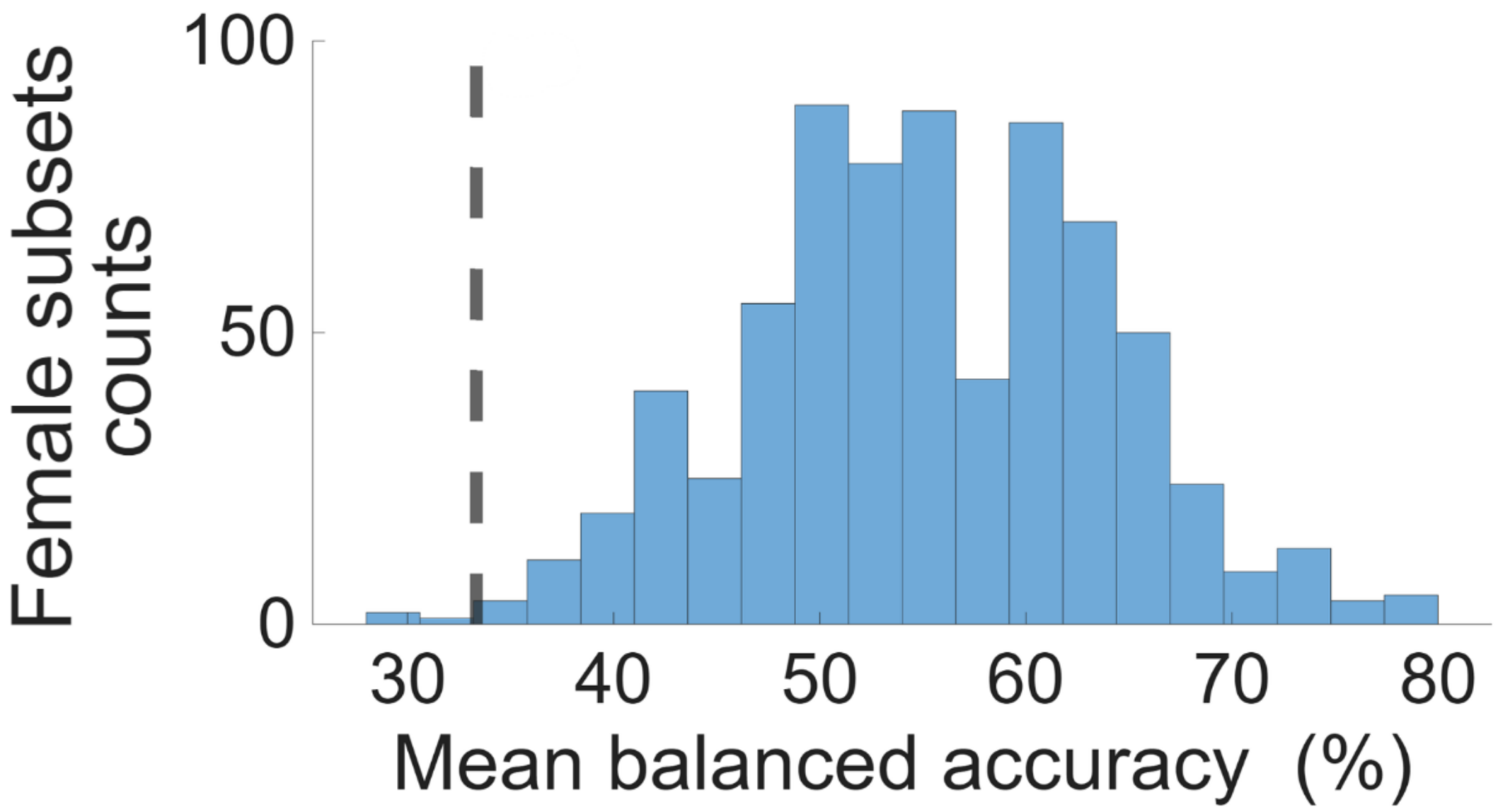

Figure S6. Sample-size-matched sensitivity analysis of fine drought discrimination. Distribution of mean balanced accuracies obtained from all 715 unique subsets of nine females selected from the 13 available female individuals (using the same data as **Fig. 4a**). The dashed vertical line indicates the mean balanced accuracy obtained from the complete group of nine males. The distribution of size-matched female performance was predominantly above the male estimate, indicating that the observed sex difference was not explained solely by unequal sample size.

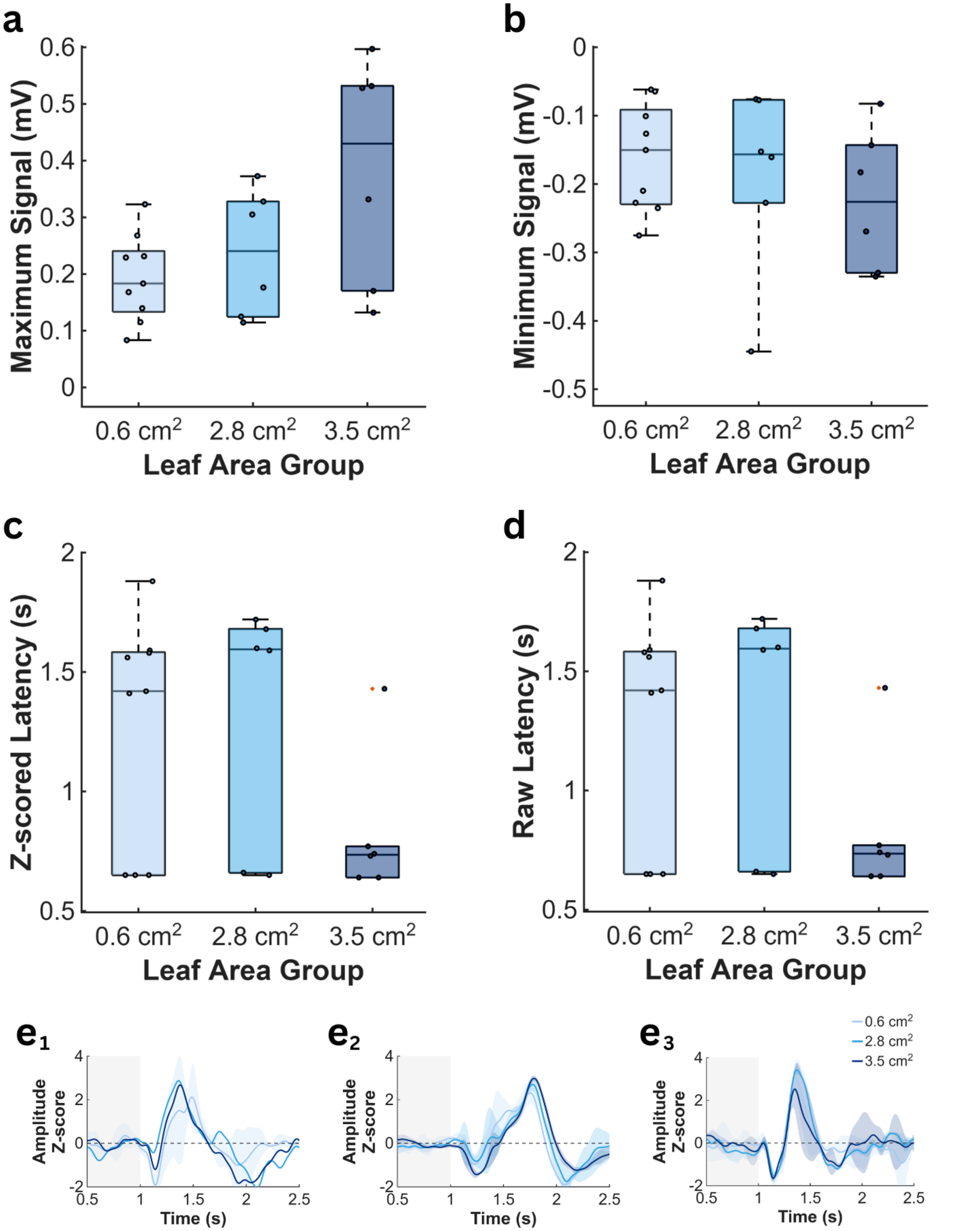
a
Maximum Signal (mV)
Leaf Area Group
0.6 cm2
2.8 cm2
3.5 cm2
b
Minimum Signal (mV)
Leaf Area Group
c
Z-scored Latency (s)
Leaf Area Group
d
Raw Latency (s)
Leaf Area Group
e1
e2
e3
Amplitude Z-score
Time (s)
0.6 cm2
2.8 cm2
3.5 cm2

Figure S7. Control experiment – The effect of tomato leaf area on EAG amplitude and temporal response dynamics. Female *Spodoptera littoralis* antennae (n = 3) were stimulated with fresh tomato leaf material (*Solanum lycopersicum*) excised from a single shoot, utilizing three measured leaf areas (0.6, 2.8, and 3.5 cm^2). Each condition was presented in triplicate per antennal preparation; recordings exhibiting a low signal-to-noise ratio were excluded from subsequent analyses. **(a, b)** Maximum (a) and minimum (b) EAG signal amplitudes derived from raw recordings. **(c, d)** Peak latencies calculated from z-score-normalized (**c**) and corresponding raw (**d**) traces. Note that z-score normalization rescales amplitude while preserving the temporal coordinates of response features, yielding consistent latency estimates regardless of the processing approach. Boxplots denote medians and interquartile ranges (IQRs), with whiskers extending to 1.5× IQR; overlaid points indicate individual mean responses. **(e_1-e_3)** Mean z-score-normalized temporal EAG profiles for individual antennae. Solid colored lines and shaded envelopes represent the mean and standard deviation across the replicates per treatment, respectively. Gray shaded regions indicate the 0.5 s odor stimulus; horizontal dashed lines denote the normalized baseline. A point-by-point permutation analysis with Family-Wise Error (FWE) max-statistic correction revealed no significant divergence in temporal profile trajectories across treatments (minimum FWE-corrected p = 0.83).

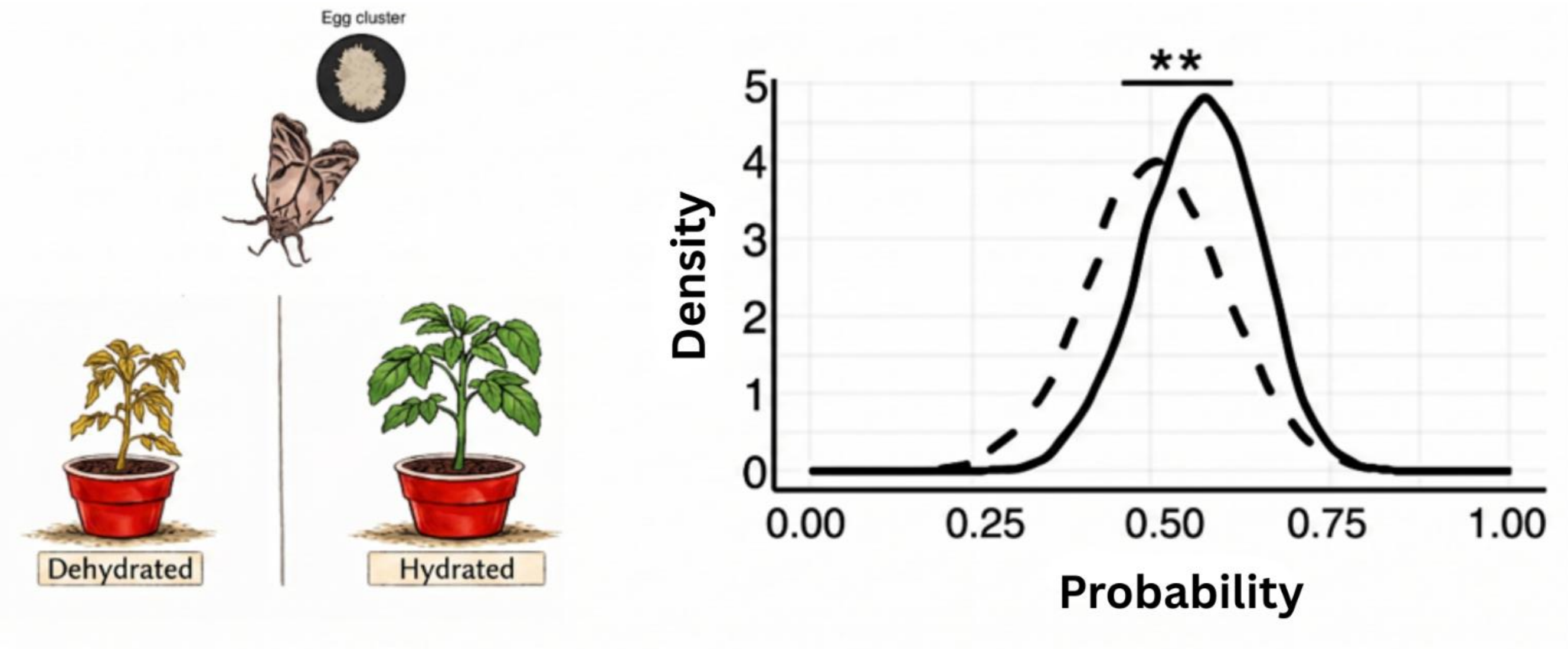

Figure S8. **Oviposition preference for hydrated versus dehydrated plants** (modified from Seltzer, Zer Eshel et al., 2026[37]). **Left**: schematic of the two-choice oviposition experiment, in which females were given overnight access to a well-watered and a dehydrated plant. Egg clusters deposited on each side were counted the following morning. **Right**: Bayesian prior (dashed) and posterior (solid) distributions for the probability of oviposition on the hydrated plant side. Egg clusters laid on the hydrated side were coded as 1, and those laid on the dehydrated side as 0. Females showed a significant preference for ovipositing on hydrated plants (hydrated: 2.23 ± 2.68; dehydrated: 0.88 ± 1.11; mean ± SE; n = 17; GLMM, $p = 0.01$).

		Mean ± SD (%)	Mean ± SD (%)	
**Class**	**Metric**	**Females**	**Males**	**F-M**
**Edible**	**Recall**	44.90 ± 2.70	37.59 ± 4.14	+7.33
**Stressed**	**Recall**	44.40 ± 3.75	54.28 ± 4.13	-9.84
**Non-host**	**Recall**	66.16 ± 3.06	53.00 ± 5.83	+13.16
**Edible**	**Precision**	67.02 ± 2.04	57.91 ± 3.59	+9.12
**Stressed**	**Precision**	29.61 ± 2.30	47.79 ± 3.04	-18.17
**Non-host**	**Precision**	50.48 ± 1.76	27.80 ± 2.87	+22.68
**Edible**	**F1-score**	53.75 ± 2.26	45.51 ± 3.84	+8.24
**Stressed**	**F1-score**	35.52 ± 2.68	50.76 ± 3.00	-15.24
**Non-host**	**F1-score**	57.25 ± 2.00	36.44 ± 3.66	+20.81

Table S1. Sex-specific classification performance across plant ecological categories. Summary of class-specific recall, precision, and F1-score (Mean ± SD (%)) for females and males moths obtained from the Gradient-Boosting classifier, along with the performance difference (F-M)
